# Composite Laser Ceramics by Advanced Bonding Technology

**DOI:** 10.3390/ma11020271

**Published:** 2018-02-09

**Authors:** Akio Ikesue, Yan Lin Aung, Tomosumi Kamimura, Sawao Honda, Yuji Iwamoto

**Affiliations:** 1 World Lab., Nagoya 456-0023, Japan; poly-yan@r2.dion.ne.jp; 2 Osaka Institute of Technology, Osaka 535-8585, Japan; tomosumi.kamimura@oit.ac.jp; 3 Nagoya Institute of Technology, Nagoya 466-8555, Japan; honda@nitech.ac.jp (S.H.); iwamoto.yuji@nitech.ac.jp (Y.I.)

**Keywords:** composite, optical ceramics, mechanical strength

## Abstract

Composites obtained by bonding materials with the same crystal structure and different chemical compositions can create new functions that do not exist in conventional concepts. We have succeeded in bonding polycrystalline YAG and Nd:YAG ceramics without any interstices at the bonding interface, and the bonding state of this composite was at the atomic level, similar to the grain boundary structure in ceramics. The mechanical strength of the bonded composite reached 278 MPa, which was not less than the strength of each host material (269 and 255 MPa). Thermal conductivity of the composite was 12.3 W/mK (theoretical value) which is intermediate between the thermal conductivities of YAG and Nd:YAG (14.1 and 10.2 W/mK, respectively). Light scattering cannot be detected at the bonding interface of the ceramic composite by laser tomography. Since the scattering coefficients of the monolithic material and the composite material formed by bonding up to 15 layers of the same materials were both 0.10%/cm, there was no occurrence of light scattering due to the bonding. In addition, it was not detected that the optical distortion and non-uniformity of the refractive index variation were caused by the bonding. An excitation light source (LD = 808 nm) was collimated to 200 μm and irradiated into a commercial 1% Nd:YAG single crystal, but fracture damage occurred at a low damage threshold of 80 kW/cm^2^. On the other hand, the same test was conducted on the bonded interface of 1% Nd:YAG-YAG composite ceramics fabricated in this study, but it was not damaged until the excitation density reached 127 kW/cm^2^. 0.6% Nd:YAG-YAG composite ceramics showed high damage resistance (up to 223 kW/cm^2^). It was concluded that composites formed by bonding polycrystalline ceramics are ideal in terms of thermo-mechanical and optical properties.

## 1. Introduction

Since the development of the ruby laser by Maiman in 1960 [1,2], other crystals—such as YAG, YVO_4_ and so on [3,4,5]—were developed as laser materials and their laser performances have been investigated. Additionally, the Nd:YAG ceramic laser gain material was successfully developed in 1995 [6,7] and, although it includes grain boundaries, efficient laser generation using this ceramic was reported. Since then, ceramic laser gain materials such as sesquioxide [8,9,10] that were difficult to grow with the conventional melt-growth method, and composite laser elements of complex design [11,12] for the creation of new laser functions, were regarded as very important materials in the field of laser engineering, especially in power scaling by large-size gain media [13].

In recent years, composite crystals formed by bonding dissimilar crystals with different chemical compositions has been suggested in order to create new laser functions [14], and excellent performance has been confirmed. Generally, precisely-polished crystals are contacted with (or without) adhesives and then heat-treated at a high temperature to produce diffusion-bonded composite crystals. It has been already confirmed that composite laser gain media give superior laser performance than their monolithic counterparts. However, there are fewer studies on the characteristics of the composite materials as well as the bonding technology and it is necessary to verify whether the composite has sufficient performance to be able to function as optics.

We have succeeded in the development of ceramic composite materials having an ideal bonding interface, which has equivalent properties to the host materials in terms of thermal conductive properties, mechanical strength properties, and scattering characteristics. This ceramic composite technology will not only be effective for future laser technology, but will also spread to the development of optics and composite manufacturing technology in other fields.

## 2. Current Status of Single Crystal Composites and Their Technical Issues

The basic concept of composite technology is to create multiple, innovative functions of lasers by combining two or more crystals with different compositions. In the conventional method for producing composite laser elements, precisely polished surfaces of crystals make contact with each other with or without adhesives and are heat-treated at a high temperature for diffusion bonding [13]. However, there are some major technical issues that must be solved to allow the development of a high-power laser system.

The first issue is in polishing technology. Generally, the surface flatness of crystal for diffusion bonding is set between λ/10 and λ/20 (λ = 633 nm). This implies that the surface has waviness (irregularity) of approximately several tens of nanometers. When crystal surfaces having this waviness are in contact with each other, interstices generate at the bonded interface, and only a partially bonded composite can be achieved. In some cases, index matching adhesives are filled into those interstices which leads to lower bonding strength.

The second issue is in the alignment of crystal orientation during the bonding process. It is impractical to completely match the crystal orientation of each crystal to be bonded. Even if it was possible to actualize the perfect surface flatness of the crystal with very precise polishing, it is impossible to align the YAG lattice of one crystal exactly onto the YAG lattice of another crystal, as it generates some kind of lattice defect at the bonding interface. Accordingly, the above two issues are still unsolved by the currently existing technology.

**Figure 1:**
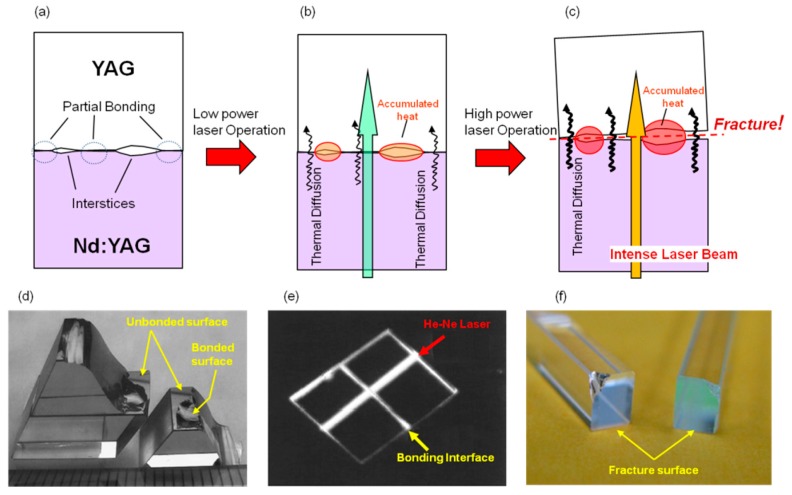
(**a**–**c**) Problem of current composite used by single crystals; (**d**,**e**) typical results from bonding between Ti:Sapphire; (**f**) fracture surface of single crystal composite after bending experiment.

In the case of crystal composites formed by currently existing technology, the bonding interface appears to be very good quality (no scattering line) to the naked eye but as shown in Figure 1a, principally several tens of nanometer interstices exist at the bonding interface. Use of crystal composites in laser operation, as shown in Figure 1b, is suitable for laser generation, but the amount of heat accumulated at the interstices becomes larger and larger with an increase of pumping energy due to poor heat dissipation efficiency at the interface, until it finally induces poor beam quality and thermal saturation of laser output power. When it is excited with further increased pumping density, as shown in Figure 1c, the heat accumulation reaches to extremely poor conditions due to insufficient heat dissipation, and the crystal composite may break down during operation. Figure 1d shows fracture surfaces of crystal composite samples composed of Ti:Sapphire crystals after impact test [15], and Figure 1e shows the scattering condition of the bonding interface (center line) detected by He-Ne laser. In the photograph of the sample after the impact test, we can confirm a part that was bonded in good condition (which appears in an irregular shape after breakage) but other parts had only a flat surfaces after breakage, which suggests that the mechanically polished surfaces were not yet bonded to each other. As we can observe a scattering line along the bonding interface, the bonding condition of such crystal composites is not sufficient in terms of mechanical strength property and optical quality. Figure 1f shows the fracture surface of a commercial crystal composite (end cap type: YAG/Yb:YAG/YAG, 6 × 6 × 80 mm) that was broken after a high-power laser operation test using an LD of 940 nm as the pumping source (500 W max output). Breakage of the crystal composite occurred at the bonding interface of YAG/Yb:YAG, which was in accordance with our anticipated issue as mentioned above. Accordingly, it is clear that the currently existing crystal composite has the above-mentioned technical issues and a breakthrough technology that can cover this problem is indispensable for the development of high power with a high beam quality laser system.

## 3. Fabrication and Inspection of Advanced Ceramic Composite

There are two different approaches to forming a ceramic composite with ideal bonding conditions. One of them is similar to the conventional crystal composite forming technology. First, surfaces of ceramic specimens to be bonded are precisely polished and then these polished surfaces are placed in contact with each other (without adhesives), and heat-treated at a high temperature for diffusion bonding [16] (hereafter, this method is called DB: Diffusion bonding). Similarly, in polishing of ceramic surfaces, the flatness is set to a level of λ/10–λ/20 (λ = 633 nm) but the significant difference from single crystal is that ceramic has numerous grain boundaries. Due to the existence of grain boundaries, a certain amount of creep (high-temperature deformation) occurs in ceramics at high temperatures, and it is possible to make the size of the interstices smaller (at a several tens of nanometers level), which exists at the interface of the composite, by applying high pressure. At the beginning of bonding, the size of interstices existing at the bonding interface is just a level of flattened interstice (waviness around several tens of nanometers) that depends upon the surface finishing quality at a precise polishing step. When it is heat-treated at higher temperatures, the flattened interstices tend to minimize its surface area due to the lowering of surface free energy at high temperatures, and the shape of the interstice transforms into an almost spherical void from a flattened and elongated gap. The diffusion coefficient (volume diffusion) of the ceramic grain (bulk) is similar to that of a single crystal, and it is not possible to remove those voids through the inside of ceramic grains. However, ceramic materials have numerous grain boundaries, and it is well known that grain boundary diffusion is about 2–4 orders of magnitude faster than the volume diffusion [17,18,19]. If the above-mentioned spherical voids are composed of vacuum or oxygen or hydrogen gas, it can be released through the grain boundaries of ceramics with fast G.B. diffusion, and finally the interstices and voids existing at the interface disappears. Therefore, it is possible to form an ideal bonding interface without any interstices. However, even if was possible to produce a composite with the DB method, interstices tend to remain at the bonding interface. Therefore, in this method, precision polishing of bonding surfaces and optimization of post heat treatments, such as HIP (hot isostatic pressing) treatment to reduce and eliminate voids completely via grain boundary diffusion, are indispensable.

**Figure 2:**
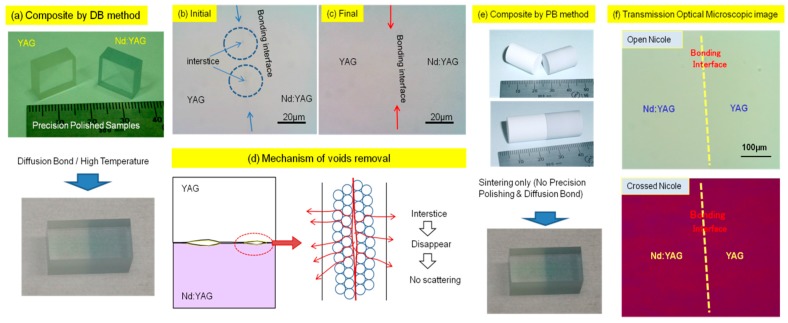
(**a**) Ceramic composite formed by DB method; (**b**) Transmission microscopic image of the composite (initial stage) after diffusion bonding with short soaking time; (**c**) final stage of the composite after HIP treatment; (**d**) Illustration of microstructure of interface from initial to final stage of bonding; (**e**) Forming YAG-Nd:YAG composite by bonding powder compacts; (**f**) bonding interface of the formed YAG/Nd:YAG composite observed under a polarized optical microscope.

Figure 2a shows a ceramic composite that was formed by precision polishing and diffusion bonding of YAG and Nd:YAG ceramics, which is similar to the fabrication of crystal composites. At the first step of bonding (initial), the polished surfaces were brought into contact with each other and heated at 1600 °C under 1 MPa, and then heat-treated again by HIP at over 49 MPa and at a higher temperature (final). Figure 2b shows a transmission microscopic image of the composite (initial stage) formed by the DB method observed near the bonding interface. Partially-formed long and narrow interstices can be confirmed along the bonding interface. Figure 2c shows a transmission microscopic image (final stage) of the composite which was heat-treated at 1750 °C for 10 h after HIP treatment at 98 MPa. The long and narrow interstices transformed into spherical shapes and these voids have been removed through the grain boundaries. The mechanism for void removal through grain boundaries and its useful effect are illustrated in Figure 2d. As a result, the scattering at the interface becomes almost zero, and the thermo-mechanical property of the composite is significantly improved. Another approach is bonding two or more powder compacts under high pressure before the sintering step as shown in Figure 2e. In this way, it is easier to produce ceramic composite laser elements as seen in the lower part of Figure 2e. Hereafter, this method is referred to as PB: Powder bonding [16]. Some of the advantages of the PB method are that precision polishing and post heat-treatments are not necessary and it also allows forming composites with curved (or irregular) shapes. Transmission optical microscopic images of the bonding interface of this composite (YAG/Nd:YAG) formed by the PB method are shown in Figure 2f. Optical stresses (double refraction) were not observed due to the difference in their chemical composition, nor were interstices. This suggests that the microstructure of the ceramic composite is not a problem for use as optics.

**Figure 3:**
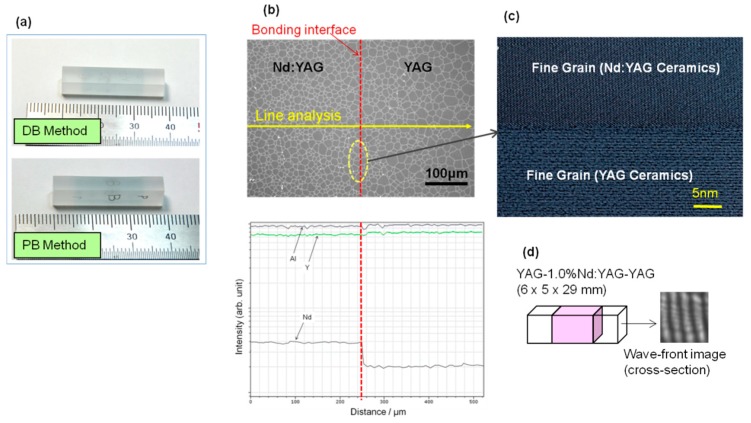
(**a**) Appearance of YAG/Nd:YAG/YAG ceramic composite formed by DB method (upper) and PB method (lower); (**b**) microstructure of bonding interface of YAG/Nd:YAG ceramic composite observed by SEM/EDX, and line analysis result for Nd ion distribution near the bonding interface by EDX; (**c**) lattice structure of bonding interface of YAG/Nd:YAG grains by HR-TEM; (**d**) wavefront image of end-capped composite by interferometer.

Figure 3a shows an appearance of ceramic composites (YAG/Nd:YAG/YAG) formed by the DB method and the PB method. In any of the composite samples, the bonding interface cannot be seen by the naked eye. SEM/EDX (scanning electron microscopy/energy dispersive X-ray, /Model SU8000, HITACHI Co., Ibaraki, Japan) analysis system was used to study the bonding interface. A result for the ceramic composite formed by the DB method is shown in Figure 3b. Interstices were not observed at the bonding interface. The grains of Nd:YAG and YAG are joint with each other and no boundary line was observed. Only a small difference in their grain sizes was observed. In the line analysis result by EDX, a sudden change of Nd ion concentration was detected at the bonding interface area but no segregation was observed at the interface. In addition, HR-TEM (high resolution transmission electron microscopy, Model 2010DM, Nippon Denshi Co., Tokyo, Japan) was used to analyze the lattice structure of the bonding interface. The result is shown in Figure 3c. Interstices were not observed at the bonding interface, and we can confirm that the bonding interface is interstice-free and the bonding is also complete at the atomic level (very similar to the typical grain boundary structure of laser ceramics). Figure 3d shows the wavefront image of an end-capped composite, which was formed by the DB method in a cross-sectional view. Straight fringes were observed, suggesting that the optical homogeneity (refractive index distribution) is very good along the direction of laser beam generation.

**Figure 4:**
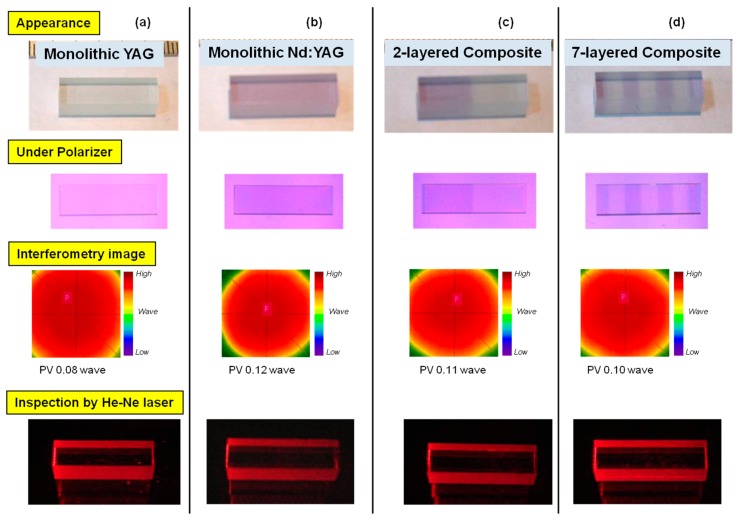
Optical properties of ceramic composites in comparison with their monolithic ceramic counterparts evaluated by polarizing plate (birefringence image), interferometry (optical homogeneity), and laser irradiation (scattering image). Measuring wavelength = 633 nm.

In Figure 4a–d, an appearance of monolithic YAG and Nd:YAG, two-layered composite and seven-layered composite formed by the PB method are shown respectively together with their polarized images, transmission wavefront images (Model F601, Fuji Film Co., Tokyo, Japan) and scattering images by He-Ne laser (λ = 633 nm) beam. In the case of monolithic YAG and Nd:YAG ceramics, double refractions were not observed, and the index distribution results by interferometry were similar to each other. Especially in the center area (an approximately 3 mm squared area) where the surface flatness is >λ/10, PV value for the YAG and Nd:YAG monolithic ceramic was λ/10 level, which was also the same in the case of two-layered and seven-layered composite ceramics. These results revealed that the optical quality of composite ceramics is as excellent as their monolithic ceramic counterparts even though the ceramic composites included many bonding interfaces. As a reference, the author prepared a single crystal composite (YAG/Nd:YAG) by a conventional precision polishing and diffusion bonding method. In this reference material (crystal composite), scattering of He-Ne laser beam can be observed at the bonding interface, suggesting that interstices remained at the bonding interface.

**Figure 5:**
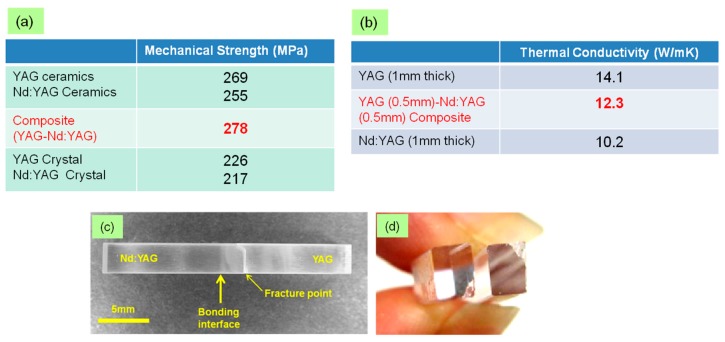

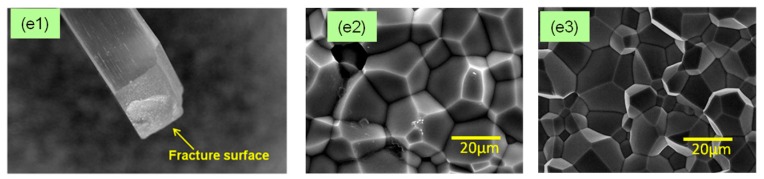
(**a**) Three point bending strength of monolithic YAG and Nd:YAG ceramics and single crystal, and YAG/Nd:YAG composite composed of polycrystalline ceramics and single crystal, appearance of YAG-Nd:YAG composite; (**b**) Thermal conductivity of YAG, Nd:YAG, and composite by laser flash method; (**c**) polycrystalline ceramics; and (**d**) commercial single crystal, after bending test; (**e1**–**e3**) magnified image of fracture surfaces of ceramic composite by stereoscope and SEM.

To evaluate the bonding condition of ceramic composites (YAG/Nd:YAG) formed by the DB method, their thermal and mechanical properties were compared with those of monolithic YAG and Nd:YAG ceramics and single crystals as well. For mechanical strength evaluation, test pieces with size of 3 × 4 × 25 mm were prepared and the four-points bending method (JIS R1601, Universal testing Machine, Model 5582, Instron Co., Massachusetts, USA) was applied. Thermal conductivity was measured by laser flash method (JIS R1611, Laser Flash Thermal Constants Analyzer, Model TC-7000, Ulvac Riko Co., Yokohama, Japan). The results from the bending test and laser flesh method are summarized in Figure 5a,b, respectively. Appearance of the ceramic composite sample after the bending test is shown in Figure 5c. (As a reference, fracture surface of the commercial crystal composite is also shown in Figure 5d.) A magnified image of the fracture surface of the ceramic composite is shown in Figure 5(e1). Fracture surface of the ceramic composite was also observed by SEM and shown in Figure 5(e2,e3). In all cases, fracture strength of ceramic composites was equal to or greater than that of their monolithic counterparts. The fracture starting point was not from the bonding interface. However, the fracture strength of the crystal composite was only about 1/10 of the host material. The fracture point was at the bonding interface, and the fracture surface was a flat surface with a mirror reflection. In the case of the ceramic composite, the fracture surface was an irregular shape and as seen in the SEM images, intergranular fracture (i.e., grain boundary fracture) was typically confirmed in any of the cases of fracture at the YAG side or Nd:YAG side. This also suggested that the fracture strength of ceramic composite is very high. Thermal conductivity of ceramic composite was just between that of the monolithic YAG and Nd:YAG. It is shown that there are no obstacles (i.e., interstices) which disturb the lattice vibration at the bonding interface, and formation of an ideal bonding condition is anticipated at the bonding interface. There are some reports on fabrication of composite elements from ceramic components [20,21]. However, due to the insufficient bonding condition, scattering at the bonding interface was significantly observed and the ceramic composite has weak points that need to be overcome.

## 4. Optical Quality of Ceramic Composite

**Figure 6:**
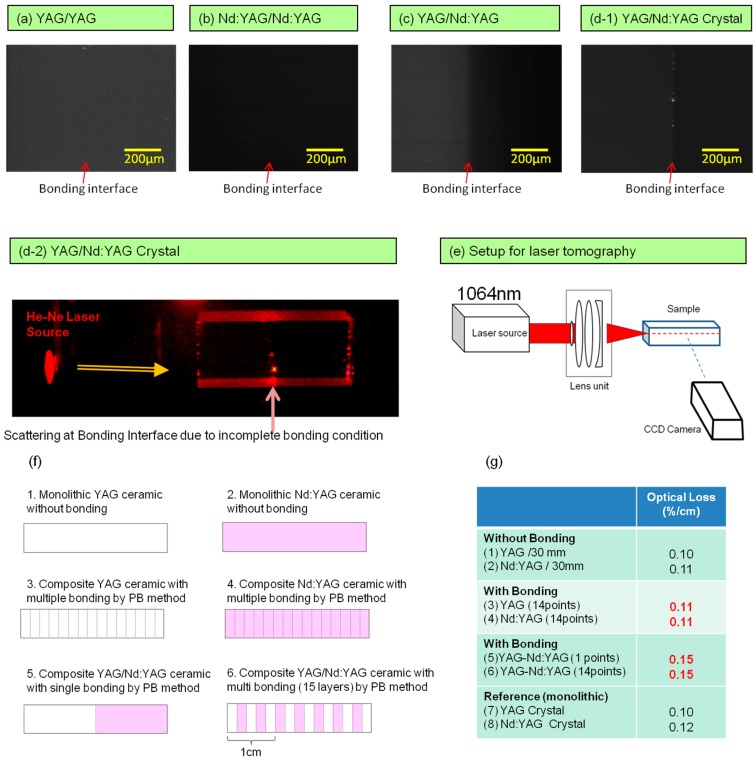
(**a**–**c**) Observation of scattering for bonding interfaces of YAG/YAG, Nd:YAG/Nd:YAG, YAG/Nd:YAG ceramic composites and (**d1**) YAG/Nd:YAG single crystal composite by laser tomography; (**d2**) scattering from heterogeneous bonding part by He-Ne laser irradiation; (**e**) schematic diagram of laser tomography using Nd:YAG laser of 1064 nm as optical source; (**f**) schematic configuration of the samples used in the measurement of optical loss; (**g**) scattering loss for (1) YAG; (2) Nd:YAG without bonding; (3) YAG; (4) Nd:YAG with joint each 2 mm; (5) YAG/Nd:YAG with one joint; (6) YAG/Nd:YAG with joint each 2 mm; (7) YAG; and (8) Nd:YAG single crystals as reference.

Laser tomography was used to measure the scattering characteristics of ceramic composites. YAG/YAG, Nd:YAG/Nd:YAG, and YAG/Nd:YAG ceramic composite samples were prepared by the PB method, and scattering measurement results on these samples are summarized in Figure 6a–c. For comparison, a result on the YAG/Nd:YAG crystal composite (DB method), which was prepared by the author, is also described in Figure 6(d1). A setup for the measurement of laser tomography is shown in Figure 6e. An Nd:YAG laser (1064 nm) was used as an optical source, and an infrared CCD camera was used to capture the scattering images. The dimension for all samples was 6 × 6 × 20 mm and a laser beam was irradiated at a 6 × 6 mm face (mirror-polished surface), and the scattering condition was observed from a 6 × 20 mm face (mirror-polished surface). In the case of ceramic composite samples formed with the same refractive index (i.e., YAG/YAG and Nd:YAG/Nd:YAG), scattering was not observed at the bonding interface as well as in the host materials. This suggests that the scattering loss of the bonding interface is extremely low (beyond the measurable level). Even when the light source was changed to visible wavelength (He-Ne laser @ 633 nm), it was difficult to observe the scattering at any position of the bonding interface. In the case of dissimilar materials (i.e., the YAG/Nd:YAG composite sample), it seems a faint scattering occurs around the bonding interface. However, in the case of the YAG/Nd:YAG crystal composite (refer to Figure 6(d1)), in addition to the faint scattering at the bonding interface, localized scattering spots can be observed along the bonding interface. Since the refractive indices of YAG and Nd:YAG are 1.80 and 1.81–1.82, respectively, scattering is theoretically due to the mismatch of refractive index occurs at the bonding interface. By laser tomography, scattering due to the index mismatch was only detected in the ceramic composite, and scattering due to other causes was beyond the measurable level. However, in the case of the single crystal composite, as shown in Figure 6(d1), when the bonding condition is very good, it was very hard to detect the scattering spots. In most of the cases, however, interstices tended to remain partially at the bonding interface and this could be easily detected by visible laser beam (He-Ne laser @ 633 nm) as shown in Figure 6(d2).

Schematic configuration of the samples used in the measurement of optical loss is shown in Figure 6f. Samples 1 (YAG) and 2 (Nd:YAG) are monolithic structures formed without bonding. Samples 3 (YAG) and 4 (Nd:YAG) are composite structures having 15 layers of the same composition (YAG or Nd:YAG) throughout the whole position of the sample formed by the PB method. Each layer was formed with a 2 mm step. Hence, they include 14 bonding interfaces in each sample. Samples 5 and 6 are also composite structures (YAG/Nd:YAG) formed by the PB method but Sample 5 has only one bonding interface of YAG and Nd:YAG, while Sample 6 has 14 bonding interfaces, in which layers of YAG and Nd:YAG composition are arranged in alternate positions. Additionally, in this composite, each layer was formed with a 2 mm step. The optical source was Nd:YAG laser (1064 nm). Surface of test samples was AR (anti-reflection) coated. The transmittance light was measured by a power meter. Optical losses measured at the 1064 nm wavelength were summarized in Figure 6g. Optical losses of Samples 1 and 2 (monolithic YAG and Nd:YAG host materials without bonding) were 0.10 and 0.11%/cm, respectively. In the case of Samples 3 and 4 (composite YAG and Nd:YAG formed by bonding 15 layers), their optical losses were 0.11 and 0.11%/cm, respectively. In this method, it was confirmed that their optical losses do not depend upon the number of bonding layers in the case of using the same refractive index materials. However, in the case of Sample 5 (YAG/Nd:YAG composite with single bonding interface), the optical loss was 0.15%/cm, which was a little bit larger than that of the host materials. In addition, in the case of Sample 6 (YAG/Nd:YAG composite formed with 15 layers), the optical loss was also 0.15%/cm, regardless of the number of bonding interfaces compared to Sample 5. The increase of optical loss in the YAG/Nd:YAG composite is attributable to the difference in refractive index (⊿n) of YAG (1.80) and Nd:YAG (1.81–1.82) at the bonding interface. In calculation, the difference in refractive index (⊿n) of about 0.01–0.02 level generates Fresnel loss of 0.1–0.2% per single bonding interface. In the case of Sample 6, there are 4 bonding interfaces of YAG/Nd:YAG at a 1 cm length. Hence, the Fresnel loss can be estimated to be 0.4–0.8%/cm (4 times 0.1–0.2%/cm) due to refractive index mismatch. Additionally, the optical loss of the host material itself was about 0.1%/cm. Therefore, the total optical loss of Sample 6 by calculation is estimated to be between 0.5–0.9%/cm. However, the measured values were very close to each other. It can be considered that the diffusion layer in the composite sample formed by the PB method is very thick (approximately 1 mm). Hence, the concentration gradient of the Nd ion (i.e., refractive index gradient) is very smooth at the bonding interface, and the scatter angle at the bonding area is so narrow that the difference in the measured values of their optical losses is apparently very small.

**Figure 7:**
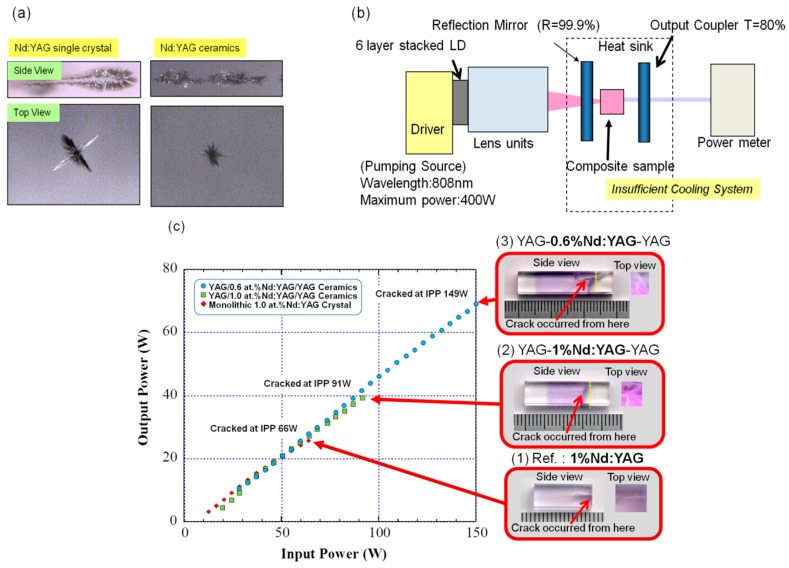
(**a**) Textures of crack for Nd:YAG single crystal and polycrystalline ceramics after irradiation of giant pulse laser with 100 J of 8 ns pulse width; (**b**) Schematic diagram for laser oscillation and damage test with insufficient cooling system; (**c**) Laser performance and cracking position of each sample.

Although the scattering at the bonding interface is very small or negligible, it is necessary to evaluate their laser damaging properties. For this measurement, the general method for LIDT (laser induced damage threshold) was applied [22]. A Nd:YAG laser was operated in a single-shot mode (at 1064 nm, 8 ns pulse duration, 7.6 GW/cm^2^ peak power) to measure the bulk LIDT. A lens with a focal length *f* = 100 mm was used to focus the laser radiation into the sample. The energy of the pulses was varied with an attenuator consisting of a polarizer and a half-wave plate. The experiments were carried out according to the ‘1-on-1’ procedure, that is, the damage threshold was measured every time on a new site of the sample. The formation of the cracks was identified by using a Nomarski microscope at a magnification of 50. Both the commercial 1% Nd:YAG single crystal and our prepared Nd:YAG ceramics were broken at around 28–31 J/cm^2^. Figure 7a showed the transmitted optical micrographs of cracks occurred inside the single crystal and ceramics observed under an optical microscope. It was confirmed that the crack size in the single crystal was larger than that in the ceramics, and the LIDT value of ceramics was larger than that of a single crystal. Fracture toughness for single crystal and polycrystalline Nd:YAG ceramics was 1.43 and 2.02 MPa·m^1/2^, respectively evaluated by JIS1607, Universal Testing Machine/Model 5582, Instron Co., MA, USA. Laser damaging is a kind of impact fracture and it is considered that the optical defects induced thermal stress and finally cracks occurred in the samples. Therefore, it is considered that the relationship of the parameters of K_IC_·κ/E·α (K_IC_: Fracture toughness, κ: Thermal conductivity, E: Elastic constant, α: Thermal expansion coefficient) is strongly correlated [23]. Apart from K_IC_, parameters are similar to each other even in single crystal or ceramics. Hence, the magnitude of K_IC_ strongly influences the laser damaging properties of ceramics.

The monolithic 1% Nd:YAG crystal (Sample 1), end-cap type YAG/1% Nd:YAG/YAG ceramic composite (Sample 2), and YAG/0.6%Nd:YAG/YAG ceramic composite (Sample 3) were prepared. The laser oscillation experiment and laser damage test were carried out on these samples at the same time in the laser oscillator shown in the Figure 7b. Sample size was 7 × 7 × 20 mm^3^, and 808 nm LD source was focused into φ200 μm. The focused beam point was irradiated at a point about 2 mm apart from the Nd:YAG/YAG bonding interface. In the case of the monolithic Nd:YAG, the focused beam was pointed to pump at a place about 2–3 mm inwards from the edge. However, in order to evaluate the damaging property during lasing action, the cooling condition was set to be insufficient. Cooling chillers (50 W) were attached on both sides (top and bottom, 30 × 80 mm) but the volume (7 × 7 × 20 mm^3^) was so large that it sufficiently released the heat from the gain medium. Figure 7c shows the points where cracks occurred and their laser performances. All of the samples showed slope efficiency of 50%. However, Sample 1, the monolithic Nd:YAG crystal, was broken at IPP (incident pumping power) 66 W(PPD/pumping power density = 80 kW/cm^2^). Sample 2 was broken at IPP = 91 W (PPD = 127 kW/cm^2^) but Sample 3 was broken at IPP = 149 W (PPD = 223 kW/cm^2^) because of lower Nd doping concentration. Noticeably, the commercial single crystal composite was broken from the bonding interface, and the very flat surface can be confirmed. The main cause of cracking was due to the insufficient cooling of the area where the pumping intensity was very high although the total cooling capacity was about 100 W by chillers. The above results suggested that the thermal mechanical properties of the ceramic composites are very similar or superior to that of the host materials. In addition, the optical loss of the bonding interface of the advanced ceramic composite is fairly small or negligible. These facts are evidence of formation of an ideal and innovative ceramic composite with ideal bonding interfaces.

## 5. Conclusions

With the advantages of ceramic bonding technology, composite laser ceramics with various designs have been demonstrated; for example, end-cap designed YAG-Er:YAG-YAG, Er:YAG with gradient doping profile, clad-core designed Yb:YAG-Cr^4+^:YAG, and so on. Optical quality and thermo-mechanical properties were found to be excellent in these structured ceramics.

Laser output with 266 W was achieved from a small scaled ceramic waveguide laser element (10 × 32 mm, YAG-Nd:YAG (400 μm core)-YAG). In addition, in the case of 0.6% Nd:YAG ceramic laser rod (Φ6 × L180 mm, bonded at every 30 mm length interval) and slab (40 × 2.5 × 120 mm, bonded at every 20 mm length interval) fabricated by this ceramic bonding technology, stable laser output power with over 4 and 7 kW levels, respectively, have been achieved in both types by end-pumping and side-pumping with 808 nm LD [24]. Details of these results will be reported in the near future. It is expected that composite ceramic laser gain media will create innovations that meet the requirements for the development of high performance and high power lasers in the future. An advanced ceramic technology that can produce ceramic laser composites with ideal bonding interfaces has drastically changed the thermo-mechanical and optical characteristics of laser gain materials. We can apply this technology not only in the fields of garnet system laser optics but also in other general optics for getting improved functionality and reliability, and also for making large-scale optics.
